# Association between service scope of primary care facilities and prevalence of high-cost population: a retrospective study in rural Guizhou, China

**DOI:** 10.1186/s12875-022-01914-5

**Published:** 2022-11-25

**Authors:** Zhong Li, Yixun Li, Ziqin Ding, Yunxi Tao, Liang Zhang, Ruibo He

**Affiliations:** 1 Institute of Healthy Jiangsu Development, Nanjing Medical University, Nanjing, Jiangsu China; 2 School of Health Policy and Management, Nanjing Medical University, Nanjing, Jiangsu China; 3 The First School of Clinical Medicine, Nanjing Medical University, Nanjing, Jiangsu China; 4 School of Pediatrics, Nanjing Medical University, Nanjing, Jiangsu China; 5 School of Political Science and Public Administration, Wuhan University, Wuhan, Hubei China; 6 School of Medicine and Health Management, Tongji Medical College, Huazhong University of Science and Technology, Wuhan, China; 7 School of Finance and Public Administration, Hubei University of Economics, Wuhan, Hubei China

**Keywords:** Service scope, Primary care facilities, High-cost population, Rural China

## Abstract

**Background:**

High-cost (HC) patients, defined as the small percentage of the population that accounts for a high proportion of health care expenditures, are a concern worldwide. Previous studies have found that the occurrence of HC population is partially preventable by providing a greater scope of primary health care services. However, no study has examined the association between the service scope of primary care facilities and the prevalence of HC populations. Therefore, this study aimed to investigate the association between the service scope of primary care facilities (PCFs) and the prevalence of HC populations within the same communities.

**Methods:**

A multistage, stratified, clustered sampling method was used to identify the service scope of PCFs as of 2017 in rural Guizhou, China. The claims data of 299,633 patients were obtained from the local information system of the New Rural Cooperation Medical Scheme. Patients were sorted by per capita inpatient medical expenditures in descending order, and the top 1%, top 5% and top 10% of patients who had incurred the highest costs were defined as the HC population. Logistic regression models were used to assess the association between the service scope of PCFs and the prevalence of the HC population.

**Results:**

Compared with those in the 95% of the sample deemed as the general population, those in the top 5% of the sample deemed as the HC population were more likely to be over the age of 30 (*P* <  0.001), to be female (*P* = 0.014) and to be referred to high-level hospitals (*P* <  0.001). After controlling for other covariates, patients who lived in the communities serviced by the PCFs with the smallest service scope were more likely to be in the top 1%, top 5% and top 10% of the HC population.

**Conclusion:**

A greater PCF service scope was associated with a reduction in the prevalence of the HC population, which would mean that providing a broader PCF service scope could reduce some preventable costs, thus reducing the prevalence of the HC population. Future policy efforts should focus on expanding the service scope of primary care providers to achieve better patient outcomes.

**Supplementary Information:**

The online version contains supplementary material available at 10.1186/s12875-022-01914-5.

## Background

High-cost (HC) populations have been widely studied worldwide. Previous studies in the United States (US) and Canada have found that the top 5% of the HC population accounts for approximately 50% of total health care expenditures [1–3]. The HC population was also defined as the top 10% or top 1% of patients by medical expenditure, who are generally most affected by chronic diseases in the last years of their lives [4]. The HC population is also often associated with high need, which means that these populations often suffer from three or more chronic diseases [1, 5]. These HC populations often require extensive attention and consume a disproportionate share of resources [6]. If their needs for health care services are unmet, they will experience a greater risk of poor health outcomes and increased health care utilization and health care expenditures [7]. Previous studies showed that the HC population status for over half of certain patients would persist for many years [8]. In China, previous studies have found that the top 5% of HC families in Hubei Province spent approximately 44.9% of the total medical expenditures on frequent emergency department (ED) visits and hospital stays [9], and approximately 68.0% of total medical expenditures of families in Jiangsu Province were attributed to the top 5% of HC patients [10].

Many studies have proven that health care costs incurred by the HC population are partially preventable. Of the 4.8% of potentially preventable Medicare spending, 73.8% was used by the HC population [11]. In 2010, 32.9% of ED-related costs were incurred by the HC population, and 41% of these costs were preventable [12]. A population-based study of HC patients with cancer in Ontario, Canada, revealed that 9% of their medical spending stemmed from potentially preventable or avoidable acute care [2]. The overuse of costly therapies and services that are disconnected from patients’ multiple needs places a heavy burden both on the patients and the health care system [13]. Previous studies have also shown that HC patients with chronic conditions rely heavily on health care services delivered by primary care providers [14, 15]. Moreover, simple interventions among patients with heart failure in outpatient settings, such as home-based physical therapy programs, may reduce unnecessary spending [11], and intensive outpatient health care programs were found to be useful in reducing ED visits, hospitalization and costs [16].

A high-quality primary care delivery system usually requires the enhanced coordination of care and a multidisciplinary team [17]. One of the representative practices is to establish specialized clinics that provide intensive medical, social and psychosocial services with a wide array of providers (nurses, medical assistants and social workers). This model has been credited with a 20% reduction in monthly health care spending and a 55% reduction in ED visits [17]. Another study found that emphasizing interdisciplinary patient engagement led to a 7% reduction in the use of hospitalization and a 31% reduction in the rate of 30-day readmission [18]. For patients with cardiovascular disease, teams with good working relationships among members were also associated with fewer hospital stays and lower costs for patients [19]. Moreover, team-based primary care services could also improve the continuity of care, and a greater continuity of care is related to lower health care expenditures [20], thus leading to a reduced prevalence of the HC population [21]. Additionally, compared to beneficiaries with fewer regular primary care visits, subgroups with more frequent primary care visits had less utilization of ED visits and hospitalization services and fewer overall Medicare costs [22].

Complex health care needs require more comprehensive and high-quality services by primary care providers [23]. However, current primary care providers in China are ill-equipped and not sufficiently trained to manage these high-need or HC populations. Many studies have shown mixed results. Compared to the non-HC group, the HC population had a lower percentage of preventable ED visits and hospitalization costs, and regions with a greater density of primary care physicians reported greater levels of preventable spending for the HC population [12]. Another study also suggested that the non-HC population had over three times the rate of preventable utilization than HC patients [24]. No consistent association was found between changes in the capacity of patient-centred medical homes and the utilization of health care services among HC patients [24]. Additionally, clinics that provide complex care management did not reduce overall expenditures after discounting upfront costs [6]. The reasons for these different results are complex. First, these interventions need substantial long-term investment, including providing the necessary resources and training courses for these providers. For example, team-based care might only provide longitudinal continuity of care rather than interpersonal continuity of care [21]. Second, the quality of these services varies widely and often cannot reach the places that need them most [5]. Third, although most studies overemphasized the utilization of acute care services, such as ED visits and hospitalization services for the HC population, nearly 70% of this spending was used in long-term care services [8], and the largest driver of inpatient spending was catastrophic events. Therefore, the factors associated with the prevalence of the HC population remain to be examined.

In China, although rural residents account for nearly half of the population, medical resources are still concentrated in urban areas [25]. To improve access to health care services and equity in the utilization of health care services, the 2009 New Health Care Reform has provided massive financial resources to provide public health care services [26], and the central government has committed to supporting innovative pilots of home or community-based care models, such as the Sanming Model and Xiamen Model [27, 28]. Although the Chinese government has made great progress in improving equal access to basic health care and financial risk protection, gaps remain in the efforts to control unreasonable increases in health care expenditures [29]. More importantly, the service scope of primary care facilities (PCFs) is narrowing, so patients tend to seek health care services at higher-level facilities [26]. Many PCFs only provide services regarding chronic disease management and close their surgical and obstetric services [30, 31]. A previous study indicated that a wider PCF service scope could reduce the utilization of health care services outside of PCFs and thus reduce overall per capita spending [26]. Therefore, it is vital to explore ways to strengthen the primary care delivery system to reduce the prevalence of the HC population. However, no studies have focused on the association between PCF service scope and the prevalence of the HC population, and the extent of the association between the service scope of PCFs and the prevalence of HC patients remains unclear. Therefore, this study aimed to investigate the association between PCF service scope and the prevalence of HC populations among corresponding communities.

## Methods

### Study design and data collection

In 2020, Guizhou Province had a population of 38.9 million within an area of 176.1 thousand square kilometres in southwestern China. In Guizhou, nearly half (46.9%) of the residents live in rural areas, with a total of 2.3 physicians and 3.0 nurses per 1000 people [32]. Per the national guidelines on the capacity-building of PCFs that were launched in 2018 [33], only 133 of the 1369 PCFs in rural Guizhou met the basic standard criteria of the national guidelines for PCFs in 2019. In other words, only 133 PCFs could provide a wider service scope of preventive and public health services in addition to basic medical care services [34].

A multistage, stratified clustered sampling method was used to collect data on the service scope of PCFs. Two cities from Guizhou were randomly selected based on their level of economic development; we then selected two counties from each city using the same principle. The detailed procedure of the sampling methods used has been described in a previous study [26]. A web-based survey using self-administered questionnaires on the service scope of PCFs in 2017 was first conducted under the coordination of the chief or deputy chief of the sampled PCFs. As some PCFs located in urban communities also served rural residents, a total of 57 rural PCFs and 7 urban PCFs were sampled. We then collected the claims data of 299,633 patients covered by these 64 PCFs from the local information system of the New Rural Cooperation Medical Scheme.

### Outcome variable

Based on the classification standards of previous studies, patients were sorted in descending order by their per capita inpatient medical care expenditures; the top 1%, top 5% and top 10% of patients who had incurred the highest costs were defined as the HC population [4, 9, 10].

### Independent variable

The facility-level service scope was divided into preventive and public health care services and basic medical care services [26]; these services are expected to be provided by the PCFs per the national guidelines for the capacity-building of PCFs that were launched in 2018 [33]. Basic medical care services consist of 1) internal medicine, 2) surgical care, 3) paediatric services, 4) gynaecology services, 5) obstetrics services, 6) dental care, 7) referee services, 8) home care, 9) telemedicine services, 10) general practice services, 11) family practice services, 12) Traditional Chinese Medicine services, 13) rehabilitation services, 14) mental health services, 15) ED services, 16) hospice care, 17) basic anaesthesiology for minor procedures, 18) medical laboratory services, 19) medical imaging services, and 20) electrocardiography services. Preventive and public health services consist of 1) residents’ health records, 2) health education, 3) vaccination, 4) health management of children aged 0–6 years, 5) maternal health care, 6) health management of older adults, 7) chronic disease management, 8) health management of patients with severe mental disorders, 9) health management of patients with tuberculosis, 10) health management services with Traditional Chinese Medicine, 11) reporting of and response to infectious disease and public health emergencies, and 12) health inspection and supervision [26]. The service scope score, ranging from 1 to 32, was calculated per cumulative service scope by PCFs [26].

### Control variables

Age group, sex, and economic status were represented by whether the patients were enrolled in the local poverty reduction scheme, whether they were reimbursed by the Critical Illness Insurance Scheme, and whether they had a referral, and these were included as covariates. Length of stay was used to represent the severity of diseases [25, 35].

### Statistical analysis

The included PCFs were categorized into five groups according to facility-level service scope. Chi-squared tests were used to compare the prevalence of the HC population among PCFs of different groups in service scope. Since a limited higher-level sample size (50 or less) could lead to biased estimates of the second-level standard errors for the two-level regression model [36] and missing values in the facility-level factors (29 of 64 PCFs did not provide us with any data on the total financial subsidies that they received from the government or their total number of staff), we used logistic regression models to assess the association between PCF service scope and the prevalence of the HC population. Multicollinearity between the included variables was assessed using the variance inflation factor (VIF > 10). In this study, the VIFs of all regression models were less than 2. Moreover, to estimate the potential unmeasured confounding necessary to explain the current results, E-values were calculated for each adjusted relative risk [37, 38]. E-values estimated what the relative risk would have to be for any unmeasured confounder to overcome the observed association between PCF service scope and the prevalence of the HC population. All analyses were conducted with Stata 14.0, and a *P* value < 0.05 was considered to be statistically significant. In addition, we performed a sensitivity analysis on the top 1%, top 5% and top 10% of patients who comprised the HC populations that had been identified using the out-of-pocket cost dataset.

## Results

### Basic characteristics

As shown in Table 1, among the 299,633 patients grouped by facility-level service scope, 92,353 (30.8%) patients were between 45 and 64 years old, and 66,805 patients (23.0%) were older than 64 years. Over half of the included patients were female (58.3%). Approximately one in six patients (13.6%) were enrolled in the local poverty reduction scheme, and 188,424 (6.2%) of them were referred. The top 5% of the HC population was more likely to be over 30 years of age (*P* <  0.001), to be female (*P* = 0.014) and to have a referral (*P* <  0.001). The top 5% of the HC population also had a slightly greater likelihood of being enrolled in the local poverty reduction scheme than the 95% general population (*P* <  0.001). No statistically significant difference in the length of stay between the top 5% HC and the 95% general population was found (*P* = 0.492). In a comparison of basic characteristics between the top 1% HC and 99% general population or the top 10% HC and 90% general population, the main results remained consistent, except for the noted difference in the sex mix between the top 1% HC and 99% general population (*P* = 0.137).

**Table 1:** Basic characteristic of enrolled patients by facilities grouped by service scope, 2017

Variable	Overall	HC (5%)	general (95%)	*P* value	HC (1%)	general (99%)	*P* value	HC (10%)	general (90%)	*P* value
Age group	299,633	14,981	284,652	**< 0.001**	2996	296,637	**< 0.001**	29,963	269,670	**< 0.001**
< 18	56,322 (18.8)	1728 (11.5)	54,594 (19.2)		315 (10.5)	56,007 (18.8)		3733 (12.5)	52,589 (19.5)	
18–29	33,030 (11.0)	1692 (11.3)	31,338 (11.0)		310 (10.4)	32,720 (11.0)		3450 (11.5)	29,580 (11.0)	
30–44	49,123 (16.4)	2932 (19.6)	46,191 (16.2)		614 (20.5)	48,509 (16.4)		5802 (19.4)	43,321 (16.1)	
45–64	92,353 (30.8)	5285 (35.3)	87,068 (30.6)		1093 (36.5)	91,260 (30.8)		10,277 (34.3)	82,076 (30.4)	
> 64	68,805 (23.0)	3344 (22.3)	65,461 (23.0)		664 (22.1)	68,141 (23.0)		6701 (22.3)	62,104 (23.0)	
Gender (%)				**0.014**			0.137			**< 0.001**
Male	125,103 (41.7)	6110 (40.8)	118,993 (41.8)		1211 (40.4)	123,892 (41.8)		12,149 (40.6)	112,954 (41.9)	
Female	174,530 (58.3)	8871 (59.2)	165,659 (58.2)		1785 (59.6)	172,745 (58.2)		17,814 (59.4)	156,716 (58.1)	
Poverty (%)				**< 0.001**			**0.041**			**< 0.001**
Yes	40,696 (13.6)	2248 (15.0)	38,448 (13.5)		445 (14.9)	40,251 (13.6)		4342 (14.5)	36,354 (13.5)	
No	258,937 (86.4)	12,733 (85.0)	246,204 (86.5)		2551 (85.1)	256,386 (86.4)		25,621 (85.5)	233,316 (86.5)	
Referral (%)				**< 0.001**			**< 0.001**			**< 0.001**
Yes	18,424 (6.2)	6144 (41.0)	12,280 (4.3)		1551 (51.8)	16,873 (5.7)		9683 (32.3)	8741 (3.2)	
No	281,209 (93.8)	8837 (59.0)	272,372 (95.7)		1445 (48.2)	279,764 (94.3)		20,280 (67.7)	260,929 (96.8)	
Length of stay*	6 (4,8)	6 (4,8)	6 (4,8)	0.492	6 (4,8)	6 (4,8)	0.838	6 (4,8)	6 (4,8)	0.193

*Note:* *, Median (p25, p75) was reported. *HC* high-cost

### Prevalence of the HC population among different PCF service scope groups

As Table 2 shows, within the top 5% HC population, we identified a gradual decline in the proportion of the HC population as the service scope of PCFs increased, which was also observed in the top 10% HC population. Although patients who lived in the communities with PCFs of the smallest service scope represented the highest proportion of the top 1% HC population and those areas that had PCFs with the greatest service scope had the lowest proportion of the top 1% HC population, the differences in the proportion of the 1% HC population between the five groups of PCF service scopes were not statistically significant (*P* = 0.173).

**Table 2:** Distribution of HC population by facility-level service scope, 2017

Categories	Quantile 1 (*N* = 58,636)		Quantile 2 (*N* = 50,539)		Quantile 3 (*N* = 81,332)		Quantile 4 (*N* = 39,637)		Quantile 5 (*N* = 69,489)		*P* value
	N (%)	%, 95 CI	N (%)	%, 95 CI	N (%)	%, 95 CI	N (%)	%, 95 CI	N (%)	%, 95 CI	
1% HC population	619 (1.06)	(0.97, 1.14)	491 (0.97)	(0.89, 1.06)	824 (1.01)	(0.94, 1.08)	414 (1.04)	(0.95, 1.15)	648 (0.93)	(0.86, 1.00)	0.173
5% HC population	3142 (5.35)	(5.17, 5.54)	2505 (4.96)	(4.77, 2.14)	4041 (4.97)	(4.82, 5.12)	1928 (4.86)	(4.65, 0.51)	3365 (4.84)	(4.68, 5.00)	< 0.001
10% HC population	6170 (10.52)	(10.28, 10.77)	4975 (9.84)	(9.59, 10.11)	8065 (9.92)	(9.71, 10.12)	3872 (9.77)	(9.48, 10.07)	6881 (9.90)	(9.68, 10.13)	< 0.001

*Note: HC* high-cost, *CI* confidence interval

### Association between PCF service scope and the prevalence of the HC population

**Fig. 1:**
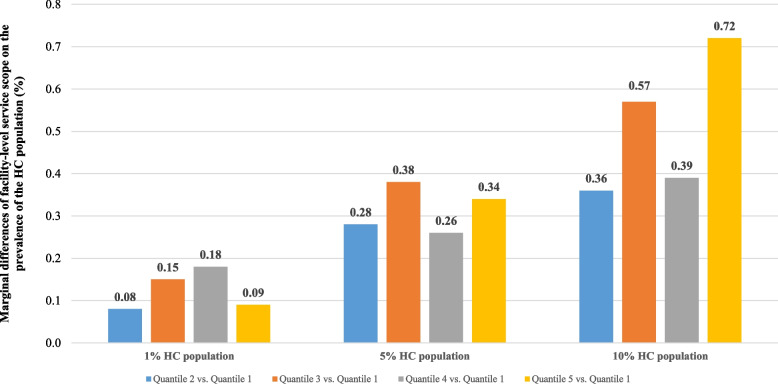
Marginal differences of facility-level service scope on the prevalence of the HC population, 2017

Figure 1 presents the marginal association between the PCF service scope and the prevalence of the top 1%, top 5% and top 10% HC populations from those same communities. In the adjusted models, compared with patients who lived in the communities with PCFs of the smallest service scope, the likelihood of there being a top 1% HC population among the populations living in communities with PCFs of greater service scope declined by 0.08, 0.15, 0.18 and 0.09%; the proportion of the top 5% HC population for patients who lived in communities with PCFs of greater service scope declined by 0.28, 0.38, 0.26, 0.34%; and the proportion of the top 10% HC population for patients who lived in communities with PCFs of greater service scope declined by 0.36, 0.57, 0.39 and 0.72% (Supplementary Table 1).

### Sensitivity analysis

**Fig. 2:**
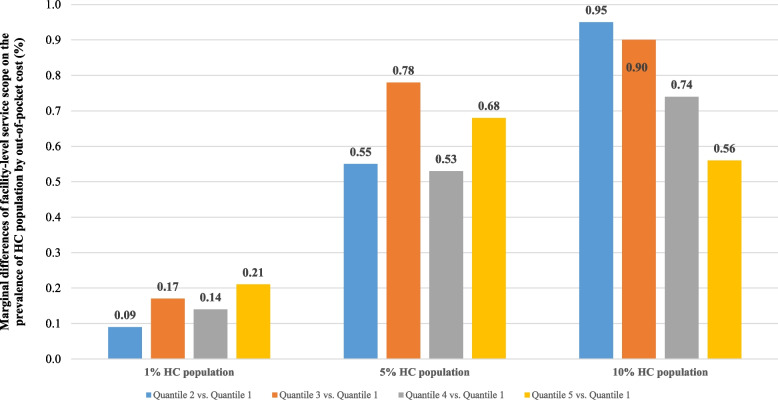
Marginal differences of facility-level service scope on the prevalence of the HC population by out-of-pocket cost, 2017

The results of regression models applied to the top 1%, top 5%, and top 10% of the HC population for out-of-pocket expenditures remained consistent with our main results both in the direction and significance (Fig. 2 and Supplementary Table 2). The E-values for the point estimates and lower 95% confidence bounds for the top 1%, top 5% and top 10% of the HC population are presented in Table 3. In this study, an E-value of 1.72 indicated that an unmeasured confounder would be necessary to increase both the likelihood of shifting from different groups of PCF service scopes and the likelihood of the top 1% HC population by 1.72-fold if PCF service scopes were not associated with the 1% HC population. In other words, the E-value of 1.72 was the minimum strength of association on the risk ratio scale that an unmeasured confounder would need to have with both the PCF service scope and the likelihood of the top 1% HC population to fully explain away the association, conditional on the measured covariates.

**Table 3:** E-values for facility-level service scope and HC population, 2017

Variable	1% HC population	5% HC population	10% HC population
	E-value (lower 95% confidence bound)		
Quantile 2 (vs. Quantile 1)	1.43 (1.00)	1.35 (1.12)	1.27 (1.07)
Quantile 3 (vs. Quantile 1)	1.63 (1.29)	1.42 (1.25)	1.37 (1.23)
Quantile 4 (vs. Quantile 1)	1.72 (1.33)	1.33 (1.04)	1.29 (1.08)
Quantile 5 (vs. Quantile 1)	1.44 (1.00)	1.39 (1.20)	1.42 (1.30)

*Note: HC* high-cost

## Discussion

To the best of our knowledge, this study is the first to examine the association between PCF service scope and the prevalence of the HC population in China. Our results not only provide evidence for potential ways to expand the service scope of primary care providers but also offer strategies for reducing the prevalence of the HC population. We found that a greater PCF service scope was associated with a lower prevalence of the HC population, which suggests that a greater PCF service scope can help avoid certain preventable costs, thus reducing the occurrence of the HC population. Differences in the proportion of HC patients by age group and sex once they had been enrolled in a poverty reduction scheme and regardless of their referral status were also observed.

First, patients living in communities with greater PCF service scopes were less likely to be in the HC population. This result may be related to the fact that an improved comprehensiveness of care can promote the use of health management services by those potential HC populations, especially those suffering from several chronic diseases [1, 5]. On the one hand, a wider PCF service scope suggests that more services are provided by primary care physicians; thus, patients’ needs would be better satisfied by primary care providers than by medical staff in high-level hospitals. On the other hand, better health management and follow-up services also lead to more utilization of preventive services that improve patients’ health status with less consumption of health care resources [39, 40]. As a result, a greater service scope can reduce the occurrence of avoidable complications, thus reducing some preventable costs. Comprehensive care could also promote interpersonal continuity of care among primary care physicians, which has been shown to be associated with a modest survival improvement without increasing the intensity of end-of-life care [41]. Given that approximately 30% of HC patients are in their last year of life [4], a greater PCF service scope might mean better care for patients during their final phase of life.

Second, we found that the marginal differences detected in facility-level service scope between the top 5% and top 10% HC populations, between the service scopes of quantile 3 vs. quantile 1 and between those of quantile 5 and quantile 1 were slightly larger than the results of comparisons between the other subgroups and quantile 1. One previous study pointed out that a greater PCF service scope could lead to a smaller rate of 30-day readmission [26], which would promote cost savings through a reduction in preventable readmissions and the unnecessary utilization of related services. We also found that older adults are more likely to be in the HC population, which is consistent with a US study that found that the HC frail and elderly group accounts for over 40% of total potentially preventable spending [4]. Moreover, a broader service scope of PCFs, combined with home-based health care services, could generate cost savings for both patients and health care providers [42]. Evidence suggests that meeting their housing, nutritional and personal care needs can also reduce patients’ health care costs [43]. However, the greatest PCF service scope was not associated with a reduction in the likelihood of the top 1% HC population, which may be related to the fact that the top 1% HC population might go to high-level hospitals directly since PCFs cannot deal with these patients’ health care needs. This result raises concerns about the effectiveness of tailored interventions that are aimed both at the population and community levels in meeting the health care needs of these HC patients, especially those in the top 1% of the HC population [44, 45].

Third, although expanding the service scope of PCFs is a promising way to meet the health care needs of the HC population and to reduce the incidence of the HC population, the presence of most intensive primary health care services did not lead to a reduced use of hospitalization services or to a reduction in their associated costs during the half-year follow-up [46]. The validation of these results through the use of a larger-scale dataset and the inclusion of a greater number of covariates, such as the travel distance between the centre of different communities and the PCFs that service them, is urgent. Additionally, policy efforts may also increase health disparities if these interventions disproportionately benefit advantaged groups while leaving vulnerable populations behind. Therefore, a more detailed assessment of the utilization of practitioner-level and facility-level services among different subgroups that could inform the capacity-building of the primary care system in China is also warranted.

## Limitations

This study has several limitations. First, the self-reported scope of service programs may be subject to social desirability bias. Second, as a less developed province in China, the fact that the findings of this study are based in Guizhou Province might limit their applicability in similar resource-limited settings. Third, different services might make different potential contributions to the prevalence of the HC population. Therefore, future studies conducted in other areas or at the national level using different weights and relying on more abundant datasets are needed to provide comprehensive evidence to better inform policy-makers, health care organizations and providers. Fourth, causal inference could be investigated based on a cross-sectional study, and future studies should conduct trials to evaluate the extent to which PCF service scope expansion could help prevent the incidence of the HC population.

## Conclusion

Our results show that a greater PCF service scope is associated with a reduction in the prevalence of the HC population, which may help inform future policies on the capacity-building of PCFs and may guide the implementation of different prevention, treatment, rehabilitation and end-of-life programs. Policy-makers could make more tailored efforts to expand the service scope of PCFs, including providing multiple primary care services and improving the ability of primary care physicians, thus collaboratively providing appropriate services and achieving better patient outcomes.

## Supplementary Information

**Additional file 1 Supplementary Table 1.** Marginal differences of facility-level service scope on the prevalence of HC population, 2017. **Supplementary 2.** Marginal differences of facility-level service scope on the prevalence of HC population by out-of-pocket cost, 2017

## Data Availability

The datasets generated and/or analyzed during the current study are not publicly available due to the authors had promised the related department that the datasets were only used for research, and they would not be disclosed, but are available from the corresponding author on reasonable request.

## Abbreviations

US: United States
HC: High-cost
PCF: Primary care facilities
VIF: Variation inflation factor
CI: Confidence Interval
