# Supplementary material for: Association between service scope of primary care facilities and prevalence of high-cost population: a retrospective study in rural Guizhou, China
Source: BMC Prim Care. 2022 Nov 25;23:301. doi: 10.1186/s12875-022-01914-5 (PMC9700956; doi:10.1186/s12875-022-01914-5)
Supplement: Supplementary file 1 — Additional file 1 Supplementary Table 1. Marginal differences of facility-level service scope on the prevalence of HC population, 2017. Supplementary 2. Marginal differences of facility-level service scope on the prevalence of HC population by out-of-pocket cost, 2017 [file 12875_2022_1914_MOESM1_ESM.docx]

**Supplementary Table 1. Marginal differences of facility-level service scope on the prevalence of HC population, 2017**

| **Variable** | **1% HC population** | **5% HC population** | **10% HC population** |
| --- | --- | --- | --- |
|  | Marginal differences (%, 95% CI) | | |
| **Quantile 2 (vs. Quantile 1)** | 0.08 (-0.03,0.20) | 0.28 (0.05,0.52) * | 0.36 (0.03,0.68) * |
| **Quantile 3 (vs. Quantile 1)** | 0.15 (0.05,0.25) ** | 0.38 (0.17,0.59) *** | 0.57 (0.28,0.86) *** |
| **Quantile 4 (vs. Quantile 1)** | 0.18 (0.06,0.30) ** | 0.26 (0.01,0.52) * | 0.39 (0.04,0.74) * |
| **Quantile 5 (vs. Quantile 1)** | 0.09 (-0.02,0.19) | 0.34 (0.12,0.56) ** | 0.72 (0.41,1.02) *** |

Note: HC, high-cost; age group, gender, poverty, referral, and length of stay were set as covariates; *, *P* < 0.05, **, *P* < 0.01; ***, *P* < 0.001.

**Supplementary 2. Marginal differences of facility-level service scope on the prevalence of HC population by out-of-pocket cost, 2017**

| **Variable** | **1% HC population** | **5% HC population** | **10% HC population** |
| --- | --- | --- | --- |
|  | Marginal differences (%, 95% CI) | | |
| **Quantile 2 (vs. Quantile 1)** | 0.09 (-0.02,0.2) | 0.55 (0.32,0.79) *** | 0.95 (0.64,1.27) *** |
| **Quantile 3 (vs. Quantile 1)** | 0.17 (0.07,0.27) ** | 0.78 (0.57,0.99) *** | 0.90 (0.62,1.17) *** |
| **Quantile 4 (vs. Quantile 1)** | 0.14 (0.02,0.26) * | 0.53 (0.27,0.78) *** | 0.74 (0.41,1.08) *** |
| **Quantile 5 (vs. Quantile 1)** | 0.21 (0.11,0.32) *** | 0.68 (0.46,0.9) *** | 0.56 (0.27,0.85) *** |

Note: HC, high-cost; age group, gender, poverty, referral, and length of stay were set as covariates; *, *P* < 0.05, **, *P* < 0.01; ***, *P* < 0.001.
